# Barriers and Facilitators for Return to Work from the Perspective of Workers with Common Mental Disorders with Short, Medium and Long-Term Sickness Absence: A Longitudinal Qualitative Study

**DOI:** 10.1007/s10926-021-10004-9

**Published:** 2021-09-27

**Authors:** Margot C. W. Joosen, Marjolein Lugtenberg, Iris Arends, Hanneke J. A. W. M. van Gestel, Benedikte Schaapveld, Berend Terluin, Jaap van Weeghel, Jac J. L. van der Klink, Evelien P. M. Brouwers

**Affiliations:** 1 Tilburg School of Social and Behavioral Sciences, Tranzo Scientific Center for Care and Welbeing, Tilburg University, Postbus 90153, 5000 LE Tilburg, The Netherlands; 2 Department of Health Sciences, Community & Occupational Medicine, University Medical Center Groningen, University of Groningen, Groningen, the Netherlands; 3 Ministerie van Buitenlandse Zaken, The Hague, Netherlands; 4 Department of General Practice, Amsterdam University Medical Centers, Amsterdam Public Health Research Institute, Amsterdam, The Netherlands; 5 Phrenos Centre of Expertise, Utrecht, The Netherlands; 6 Optentia, North West University of South Africa, Vanderbijlpark, South Africa

**Keywords:** Mental health, Work disability prevention, Sick leave, Return-to-work, Workers’ perspective, Barriers and facilitators

## Abstract

*Purpose* Although common mental disorders (CMDs) highly impact individuals and society, a knowledge gap exists on how sickness absence can be prevented in workers with CMDs. This study explores: (1) workers’ perceived causes of sickness absence; (2) perceived return to work (RTW) barriers and facilitators; and (3) differences between workers with short, medium and long-term sickness absence. *Methods* A longitudinal qualitative study was conducted involving 34 workers with CMDs. Semi-structured interviews were held at two time-points during their RTW process. The 68 interviews were audio-taped, transcribed and thematically analyzed to explore workers’ perspective on sickness absence causes, RTW barriers and facilitators, and compare data across the three sub-groups of workers. *Results* Workers reported various causes for their absence, including: (1) high work pressure; (2) poor work relationships; (3) unhelpful thoughts and feelings, e.g. lacking self-insight; and (4) ineffective coping behaviors. According to workers, RTW was facilitated by work adjustments, fulfilling relationships with supervisors, and adequate occupational health guidance. Workers with short-term leave more often reported favorable work conditions, and proactive coping behavior. In contrast, the long-term group reported reactive coping behavior and dissatisfaction with their work. *Conclusion* Supporting workers with CMDs in gaining self-awareness and regaining control, discussing the value of their work, and creating work conditions that enable workers to do valuable work, seem central for successful RTW and might prevent sickness absence. Supervisors play a key role in enabling workers to do valuable work and further research should focus on how supervisors can be supported in this task.

**Supplementary Information:**

The online version contains supplementary material available at 10.1007/s10926-021-10004-9.

## Introduction

Despite the increased attention for long-term sickness absence in workers with common mental disorders (CMDs), such as depression, anxiety and adjustment disorders, work disability associated with CMDs has significantly increased over the last decade and is one of the leading causes of sickness absence and long-term work disability in industrialized countries [1, 2]. Given the high associated costs (i.e. sickness benefits, lost productivity) and reduced quality of life of workers, CMDs have a major impact on individuals and society [3].

Over the past decades, several studies have shown that multicomponent interventions, which include work-focused elements, are promising in facilitating earlier RTW in workers with CMD [4–6]. Also, studies have provided new directions for future research that have increased our understanding of the antecedents of CMD-related sickness absence. First, studies have shown the importance of workers’ attitudes towards RTW, as these affect actual RTW [7–11]. Moreover, it has been argued that for sustainable employment, a focus on the individual worker’s values and capabilities is of importance [12, 13]. Here, ‘values’ refer to what a worker finds important in work (e.g. increase knowledge and skills). Capabilities refer to whether the worker is enabled by the work environment (e.g. provided with learning opportunities) and being able (e.g. motivated and assertive) to realize this.

Second, some authors have suggested that RTW should no longer be seen as a one-time event but should be measured as a *process* during which there may be several stages, and where crucial influences may change over time [14–16]. Workers’ thoughts, feelings and behavior about the past, present, and future, in relation to RTW are likely to change over time. For instance, during times of high stress at work, workers’ views on work pressure and work relationships may be different from their retrospective views after a few months of rest, being at home on sick leave. To date, there is limit knowledge on the RTW process, including what happens during the sickness absence process and after initial work resumption [17]. To study this, an in-depth qualitative research approach has been recommended [14].

Third, research has shown that workers with CMDs on sickness absence are not one homogenous group, but show different patterns in symptoms recovery, RTW trajectories and sickness absence duration [14, 15, 18–20]. Researchers should focus on subgroups, such as short- versus long-term sick leave, to which specific interventions can be targeted. Specific knowledge on subgroups can contribute to the development of personalized RTW guidance which may help to prevent long-term absenteeism.

In order to gain more insight into how long-term absence (i.e. absent for more than 6 months) in workers with CMDs can be reduced and prevented, the aforementioned three new research insights were incorporated in the present study. Specifically, the research questions were:

1. What do workers with CMDs on sick leave perceive as causes for their sickness absence?
2. What do workers with CMDs on sick leave perceive as barriers and facilitators for their RTW during their RTW process?
3. What are differences and similarities in perceptions between workers with CMDs with short (< 3 months), medium (3–6 months) and long-term (> 6 months) sickness absence?

## Method

### Study Design

A qualitative longitudinal design using face-to-face interviews was chosen as it enabled us to obtain in-depth information about behavior, underlying motivation, needs and preferences of the target group [21]. Semi-structured face-to-face interviews were conducted with workers at two time-points during their RTW process. The first phase of interviews was held shortly after the start of the sickness absence period. The second phase was held after workers had resumed work (short and medium-term sickness absence group), or after 6 months (long-term sickness absence group).

Prior to the start of the study, ethical approval for this study was obtained from the Ethics Review Board (ERB) of the School of Social and Behavioral Sciences of Tilburg University (EC-2015.32). All participating workers gave their written informed consent prior to the interviews. The COREQ guidelines [22] were used in the design and reporting of this study.

### Setting

This study was conducted in the Netherlands. According to the Dutch Gatekeeper Improvement Act [23, 24] the employer is responsible for the RTW process of sick-listed workers during the first two years of sickness absence. During the absence period the employer is obliged by law to continue paying wages (at least 70%), irrespective of cause and work-relatedness. During these two years the sick-listed worker cannot be fired. The occupational physician (OP) has a central role in the Dutch social security system. An OP is a qualified medical doctor specialized in occupational health who assists employers and workers in occupational health issues, safety and sickness absence management. The employer has to provide access to an OP within 6 weeks of sickness absence of the worker. OPs work for an Occupational Health Services (OHSs) or are operating independently and are contracted by employers for their services.

### Participants and Recruitment

Workers on sick leave were invited to participate by (occupational) health professionals, such as occupational physicians, psychologists, general physicians, who supported workers with CMD. These professionals were identified via the researchers’ network, social media and websites of (mental) healthcare organisations. If workers gave permission, the worker’s contact details were shared with the researchers who contacted the worker by email of phone to check for eligibility. Inclusion criteria were: (1) sickness absence for a maximum of 6 weeks at the start of recruitment, of which the worker was at least 1 week fully absent from work, (2) aged 18–65, (3) Dutch speaking, and (4) mental health issues were the primary reason for the worker’s sickness absence according to the professional. Excluded were workers who were suicidal. Potential participants received a leaflet per email containing information about the study and were given the opportunity to ask questions through email or by telephone.

If workers were willing to participate, a brief psychiatric interview (i.e. Primary Care Evaluation of Mental Disorders (PRIME-MD) [25] by telephone was scheduled and an appointment was made for the first interview at a time and location that was most convenient to the participant (mostly their home). After the first interview, the researcher emailed the participating workers every two weeks to monitor their current sickness absence and work status. Based on their pace of returning to work three groups of workers were distinguished: (1) workers who resumed within 3 months (short-term group), (2) workers who resumed within 3–6 months (medium-term group), and (3) workers who had not resumed work after 6 months (long-term group) [26, 27].

For this prospective study, saturation (defined as when no new concepts emerge from the data) needed to be reached for all three subgroups after the second phase (see also analysis). As the absence duration of each worker was still unclear during the first interview, a relatively high number of workers had to be interviewed during the first phase. During the second phase, most participants appeared to belong to the short and medium-term group, where saturation was quickly reached. However, it was more difficult to identify sufficient workers for the long-term group. Therefore, not all respondents who participated in the first interview were included in the study (i.e. they were not interviewed a second time, and their data of the first interview were not analysed). For the second phase interviews, a selection of workers who participated in the first phase was made, based on group type and workers’ professions. After 12 interviews in the short-term group, 11 in the medium-term and 11 in the long-term group, saturation was reached in all three groups.

### Data Collection

The interviews were held by one of three trained researchers (HvG, BS, EB). The interviews started with a short conversation and introduction of the interview focus. Participants were reassured that there were no right or wrong answers and that the interview data would be handled confidentially. Prior to the start of the interview, participants provided their written informed consent. All interviews were audio-taped, with permission of the participants. After each interview participants received a gift voucher of 10 euros.

A semi-structured topic guide was used for both phases of interviews, which was developed based on relevant literature and expert opinion from the project team (See Online Appendix). The first phase interviews focused on workers’ views on the causes of their sickness absence and expectations concerning barriers to and facilitators of work resumption. The main topics of the second phase interviews were how the workers had experienced their sick leave period and RTW process, and which factors they perceived as hindering or facilitating for the RTW process.

### Data Analysis

All 68 transcripts of the interviews among 34 workers were transcribed verbatim and all identifying characteristics were removed. A thematic analysis strategy was deployed [28], using the software program AtlasTi version 7.5.16. The research questions were used as framework for the analyses, i.e. perceived causes of sickness absence, barriers and facilitating factors for RTW.

First, three transcripts were independently (openly) coded by HvG and BS. These initial codes were compared and discussed and a preliminary coding scheme was generated. Next, seven additional transcripts were independently coded by HvG and BS using the preliminary coding scheme. The remaining 58 transcripts were coded by one researcher (HvG, ML, MJ or BS) using this coding scheme and was checked by a second researcher (BS or ML). Code agreements and disagreements were discussed until consensus was reached.

After all data were initially coded, all codes were sorted into potential themes. Two researchers (ML, BS) identified relations between codes and organised them into categories and subcategories per research question (i.e. causes of sickness absence, RTW barriers and facilitators). This coding scheme was discussed with a third researcher (MJ) until consensus was reached again.

The next step consisted of reviewing and refining the set of candidate themes by discussions with the research team, followed by checking them in accordance to the complete data set. This resulted in the identification of core categories of perceived causes and perceived RTW barriers and facilitators. For analysing the similarities and differences between the subgroups, the themes of both causes of sickness absence and RTW barriers and facilitators within each subgroup of workers were discussed and compared in the multidisciplinary research team. Based on these comparisons, the team interpreted and formulated differences and similarities between the three subgroups.

Constant comparison was applied throughout the whole process of data analysis, by comparing the emerging themes with new data, across individuals, and across different groups of workers. Saturation was reached after no new concepts emerged from the data in all three subgroups. Inter-observer reliability was tested on several occasions, through coding of the first ten transcripts by more than one coder and by organising several group discussions (on meaning) of codes and relationships between codes.

## Results

In Table 1, characteristics of the 34 workers are shown; they reported various (and sometimes multiple) mental health conditions, with the most prevalent PRIME-MD diagnoses being major depressive disorder (n = 19), somatoform disorder NOS (n = 13), and generalized anxiety disorder (n = 11). No clear differences were found regarding the severity or type of disorders per group. Participants had various job roles (e.g. managerial, health, education, manual labor).

**Table 1:** Characteristics of participating workers with CMDs

	Participants			
	Short-term sick leave group (< 3 months)	Medium-term sick leave group (3–6 months)	Long-term sick leave group (> 6 months)	Total
Number of participants	12	11	11	34 (100%)
Sex				
Male	5	2	1	8 (23,5%)
Female	7	9	10	26 (76,5%)
Mean Age in years (range)	48 (29–59)	48 (37–60)	52 (40–62)	49 (29–62)
Educational level				
Low [primary school]	2	1	0	3
Middle [secondary education]	4	6	2	12
High [vocational education or university]	6	4	9	19
Disorders (number of participants)^a^				
* Any psychiatric disorder*	12	9	9	30
* Any mood disorder*	11	8	7	26
Minor depressive disorder	1	0	1	2
Major depressive disorder	7	6	6	19
Partial remission or recurrence of major depressive disorder	2	2	1	5
Dysthymia	3	2	3	8
R/O bipolar disorder	2	2	0	4
*Any anxiety disorder*	7	7	6	20
Panic disorder	0	0	1	1
Anxiety disorder NOS^b^	3	3	3	9
Generalised anxiety disorder	5	4	2	11
*Probable alcohol abuse/dependence*	0	0	0	0
*Any eating disorder*	1	0	0	1
Bulimia nervosa, ‘purging type’	0	0	0	0
Bulimia nervosa, ‘nonpurging’ type 2	0	0	0	0
Binge eating disorder	1	0	0	1
*Any somatoform disorder*	7	8	6	21
Multi-somatoform disorder	3	2	3	8
Somatoform disorder NOS^a^	4	6	3	13

^a^Measured with the PRIME-MD [25]

^b^*NOS* not otherwise specified

### Perceived Causes of Sickness Absence

Seven main themes and 26 subthemes in terms of perceived causes of sickness absence were identified (Box 1). Four main themes were identified as most prominent:

(I) High Work Pressure and Other Unwanted Changes in Work
  An excessive workload or work pressure was often reported as the primary reason for workers’ sickness absence. According to many workers, their sickness absence was preceded by an extended period of time where their workload or work pressure had been too high, eventually leading to an emotional and sometimes also physical breakdown. A combination of two types of reported high workload were identified: an ‘objective’ description of a work environment with high work pressure (e.g. working additional hours, tight deadlines), and a ‘subjective’ description of high work pressure that seemed to be self-imposed. Here, respondents indicated to work harder than strictly necessary, out of perfectionism, the inability to be assertive and a high sense of responsibility. Workers often mentioned that a combination of these two types of workload (which could exist together) seemed to be the primary cause for their sickness absence.
  I also think that that has been the reason that I broke down on this, because I wanted to do it all too well, the feeling of responsibility. And despite the fact that I just didn’t have the time and I couldn’t get it all together, I still wanted it and felt responsible. [woman, 44 years, team leader]
  A recurrent subtheme was that respondents felt their work content and tasks had changed over time and became less fulfilling for them. For instance, additional administrative and computer tasks that had accumulated over the years were often reported as a nuisance by workers.
  When I started twelve years ago versus now… Now records need to be kept of everything, you have to meet a lot of requirements, […] you have to take notes of everything, do training, all the extra things. Well, I think that is what ruined me. […] And the endless flow of ICT and when I understood the ICT then one year later it was different again. And I couldn’t keep up... [woman, 62 years, teacher secondary education]

**Box 1:** Causes of sickness absence, from the perspective of workers with CMDs

**A. High work pressure and other unwanted changes at work**
A1. Changes in type of work (more tasks, different content, person-job misfit)
A2. Lack of clarity of the work content (e.g. tasks) or role at work
A3. High workload, working overtime, travel time and irregular working hours
A4. Lack of control opportunities/lack of opportunities to realise modifications at work
A5. Difficulties in adapting to technology
**B. Inadequate management and/or policy**
B1. Insufficient communication with management, inadequate policy
B2. Insufficient opportunities for training and education
B3. Experienced injustice/not feeling heard
B4. Non-fitting workplace culture
**C. Poor relationship with supervisor and/or colleagues**
C1. Lack of support by supervisor, insufficient supervision
C2. Lack of support and interaction with colleagues
C3. Bullying
C4. Experienced injustice/not feeling heard
**D. Unhelpful thoughts and feelings**
D1. High sense of responsibility
D2. Lack of self-insight/not able to observe limits
D3. Not willing to give up control
D4. Lack of self-confidence
D5. Negative perception of mental health problems
D6. Opinion and expectations of close others
D7. Situation conflicts with own values and norms
**E. Ineffective coping and behaviour**
E1. Avoidant coping
E2. Not being able to set limits/not listening to signals
**F. Complaints**
F1. Emotional and physical breakdown
**G. Home/work interference**
G1. (Informal) care giver responsibilities in private life
G2. Tensions/stress in private life
G3. Work-home imbalance
(II) Poor Relationship with Supervisor and/or Colleagues
  An important factor contributing to workers’ sickness absence was as a perceived poor relationship with and lack of support from the supervisor. Also lack of support from co-workers was mentioned frequently by workers. In addition, *experienced injustice* and *not feeling heard* by supervisor and colleagues were identified as relevant factors:
  Not being heard in a meeting... I think nothing is as bad […], as when they downplay things. Because we experience a very high work pressure […] but then when you indicate that, it is always being downplayed […]. It is really unpleasant if they don’t listen. [woman, 51 years, nurse]
(III) Unhelpful Thoughts and Feelings
  Another commonly reported subtheme was that workers indicated to have unhelpful thoughts and feelings, such as a high sense of responsibility, a lack of self-insight, and a lack of self-confidence. As a result, they tended to overlook early warning signals (e.g. headache and fatigue), were not able to observe boundaries, and/or were not willing to give up. Prior to the sickness absence, they had often been insufficiently aware that work was undermining their health and wellbeing, and was taking its toll on them.
  Looking back, there is a reason that I got burned-out and my colleague didn’t. […] I think that I am very insecure and because of that, I can’t guard my limits. […] I also have a terribly misplaced sense of responsibility and I am a real perfectionist. So even if I think my work is not really interesting, I still want to do it as good as possible and preferably perfect. [woman, 44 years, coordinator quality department]
  The vast majority of respondents reported fatigue, feeling worn out and being in need of rest. Workers also commonly reported that there had been a triggering event that was followed by an emotional or physical breakdown. After this moment they felt incapable to cope and unable to work.
  I felt bad inside, and the telephone ringing and um…I was thinking: I am going to throw that thing against the wall and… I am normally not like that. [..] [man, 52 years, team manager]
(IV) Ineffective Coping and Behaviour
  Even if workers were aware of physical or emotional signs of stress, they mentioned that their non-assertive behaviour and the inability to set boundaries often led to avoidant coping behaviours (e.g. continue working under stressful circumstances). These ineffective coping strategies in reducing stress frequently led to a break down. Despite the fact that workers felt ashamed, they felt like they had no choice but to call in sick.
  I feel ashamed, [..] because I have broken down. I have always been someone who solved her own problems and especially didn’t bother others. And I wasn’t able to do so anymore. So that was really hard for me.[…] That I needed help [woman, 53 years, operating room assistant]
  Whereas the workers acknowledged that they had mental health issues, none of the workers indicated that these were the primary cause of their sick leave. Instead, they mostly viewed *high work pressure and other unwanted changes* as the cause, and their mental health issues as a *consequence*.

## Perceived Barriers and Facilitators for RTW

### Barriers for RTW

As can be seen from Box 2, nine themes and 26 subthemes in terms of barriers to RTW were found. The three most prominent main themes were:

(I) Inadequate or Insufficient Work Adjustments
  Workers mentioned that if no changes (e.g. in work pressure or tasks) were realized in their work situation to which they were to return, this formed a major barrier to RTW. This not only referred to temporary work adjustments, but was also related to other unwanted changes (such as additional administrative tasks) for which reason they did not really enjoy their work anymore. This hindered them from returning to their job.
(II) Inadequate Management and/or RTW Policy
  Within this theme, workers mentioned a lack of support and guidance from occupational physicians, other occupational health professionals, and their employer. Workers often felt pressured by them to RTW when they felt not ready yet and mentioned having to start-up too fast, or having to work more hours than agreed upon. Other barriers mentioned were discontinuity in occupational health care, lack of follow-up contact moments, and insufficient guidance.
(III) Poor Relationships with Supervisors and/or Colleagues
  Managers’ lack of understanding and interest in the workers’ situation made workers feel angry and disappointed, which in turn was seen as a barrier to RTW. Moreover, negative responses or a lack of understanding from colleagues was also reported as a barrier to RTW, especially considering this was already a sensitive topic, because many workers felt guilty and ashamed about calling in sick. Also the feeling that the employer wanted to get rid of the employee was reported as a barrier to RTW.

**Box 2:** RTW barriers, from the perspective of workers with CMDs

**A. Inadequate or insufficient work adjustments**
A1. Non-fitting work content (tasks not challenging, person-job misfit)
A2. Non-fitting work context (workload, working hours
A3. No suitable work accommodations: no structural solutions
**B. Inadequate management and/or policy**
B1. Having to start-up too fast, no accommodations arranged
B2. Higher workload than agreed on, extra work
B3. Insufficient guidance/supervision: lack of clarity of RTW process
B4. Experienced injustice/no appreciation/lack of trust
B5. Person-organization misfit: related to work culture and worker’s norms and values
**C. Poor relationship with supervisors and/or colleagues**
C1. Lack of support, lack of understanding, inadequate communication, not approachable supervisor
C2. Lack of support and interest/empathy/understanding of colleagues
C3. Pressure from supervisor, bullying
C4. Experienced injustice/no appreciation
**D. Workers’ motivation and emotions**
D1. Lack of motivation to return to work
D2. Feeling insecure, fear of returning, fear of negative responses at work
D3. Feelings of shame or guilt
**E. Workers’ thoughts and behaviour**
E1. Not able to set limits
E2. High sense of responsibility
E3. Lack of self-insight
E4. Non-assertiveness
**F. Complaints**
F1. Physical complaints
F2. Emotional complaints
**G. Private life context**
G1. Tensions/stress in private life
G2. Lack of understanding from close others
**H. Professional guidance**
H1. Long waiting lists in treatment paths and setting diagnosis
**I. Societal factors**
I1. Legal arrangements: accommodations only possible after sickness notice

### Facilitators for RTW

Seven main themes and 23 subthemes were identified as facilitators of RTW (see Box 3), of which four main themes seemed most important:

(I) *Structural Work Adaptations*
  Work adjustments were found very helpful for RTW. Adjusting tasks or content of the work, such as temporarily working fewer hours were mentioned. But also improving working conditions, such as eliminating job strain or fewer stimuli at work (e.g. no radio or phone calls during work) were found helpful.
(II) *Adequate Management, Policy and Supervision/Guidance*
  Occupational health professionals and employers who provided structure and clarity, and managed expectations about the RTW process were mentioned as a facilitating factor for RTW. Workers also felt it was important not to be pushed to return and to get decision authority in the RTW process, so they could start working at their own pace.
(III) *Supporting Relationship with Supervisor and/or Colleagues*
  A good relationship and support from the supervisor and colleagues was a commonly reported theme. Workers mentioned that feeling safe and supported at work was helpful to return. This especially counted for support from the manager. According to workers, it felt important that the manager listened, accepted the person for who he/she is and showed understanding. Also managers could help by guiding the worker in setting their limits.
(IV) *Effective Worker Behaviour*
  As fatigue was a commonly reported complaint, workers mentioned they needed rest and to be away from work for some time. An active coping style (e.g. keeping daily structure, physical exercise) with a focus on recovery was mentioned as a facilitator for RTW. Moreover, talking about their struggles in general and explaining their situation to their colleagues and supervisor helped respondents in the RTW process. Some respondents had disclosed their situation to colleagues and felt supported by them. Also, getting more insight into their situation and to get perspective by talking to family and friends was reported as helpful.

**Box 3:** RTW facilitators, from the perspective of workers with CMDs

**A. Work adaptations**
A1. Structural or temporary work adjustments
A2. Structural changes in the work pressure/context (less job strain, fewer working hours, fewer stimuli)
**B. Adequate management, policy and supervision/guidance**
B1. No pressure on the return, enable work accommodations
B2. An active reintegration policy
B3. Supervision/guidance and communication: providing structure and clarity, managing expectations
B4. Supervisor’s appreciation for the worker’s situation
B5. Opportunity to get decision authority
**C. Fulfilling relationship with supervisor and/or colleagues**
C1. Support from supervisor
C2. Interest/empathy and support from colleagues
C3. Being accepted/feeling safe at work
C4. Sharing responsibilities with co-workers
**D. Helpful thoughts and motivation**
D1. Acceptance and self-reflection
D2. High sense of responsibility
D3. Exploring the value of work, having perspective (other job)
**E. Effective behaviour: what the worker can do**
E1. Disclosure and explain situation to work environment
E2. Take time to recover: taking rest and keeping distance from work
E3. Actively focus on recovery: keep a daily structure and continue to be active
E4. Keep in contact with work
E5. Guard limits and regain/remain in control
**F. Private life context**
F1. Work-home balance
F2. Accommodations in private life
F3. Support from family and friends
**G. Professional guidance**
G1. Professional support, guidance from a coach

### Differences and Similarities Between Workers with Short, Medium, and Long-Term Sickness Absence

When comparing the three groups, similar perspectives on sickness absence and the RTW process including the barriers and facilitators emerged. Especially the combination between high ‘objective’ work pressure and high self-imposed work pressure, and the inability to see and respond to early stress related warning signs were common in all groups. However, three main differences were found when comparing the data of the three groups, which were most noticeable between the short-term and the long-term group. These were:

I) Favourable versus unfavourable working conditions

In the short-term group, many respondents reported *favourable work conditions*, including good relationships with supervisors and colleagues in combination with a high worker motivation. They often indicated they simply had worked too hard and had ignored signs for too long, because they mentioned being workaholics, perfectionists or because they really enjoyed their jobs. In contrast, workers with long-term sickness absence experienced unfavourable working conditions mostly related to relationship with and lack of support from the manager, such as insufficient understanding, and inadequate support to adjust the work to their needs. This in turn did also not motivate the worker to return.

[What helped is] that they [manager] didn’t pressure me to return. Even though I thought I could start sooner, they said to just take it easy. And I think that’s a good thing. [Worker with short-term sickness absence: man, 56 years, head of finance]

Well, what would make it difficult…if I would return and nothing has changed, that wouldn’t be difficult, that would be impossible. But what would make it easier is if they [manager] would listen to what I say […]. And not only listen, but also do something with it. [Worker with long-term sickness absence: woman, 48 years, manager]

II) Proactive versus reactive recovery behavior

Workers in the short-term group indicated to use more *proactive recovery behaviour*. They took action to arrange whatever they needed for their recovery, such as appointments with psychologists and occupational physicians, making a structured day planning with physical exercise and social contacts, or looking for a new job. Workers with long-term sickness absence seemed to be more reactive and in need of professional guidance and help with mirroring and getting insight into what costs energy and what are energisers.

Everything that had to be arranged, I organised it myself. I mean, I organised everything – the psychotherapist, the registration etc. – myself. I made the appointment [with the occupational physician] myself. You can’t have a more exemplary patient. [Worker with short-term sickness absence: woman, 44 years, manager]

[What would have helped], I think something of guidance. Someone who talks you through the steps, but who is also your safety net. […] Someone who can hold up a mirror, because you don’t exactly have a right image of yourself and haven’t made the right choices. [Worker with long-term sickness absence: woman, 42 years, team manager]

*III) Being able to do work that one values, or not*

Workers with short-term sickness absence often indicated to like many aspects of their work (e.g. work content or social contacts at work) and had a pleasant working environment, which contributed to their RTW. In contrast, workers in the long-term group often indicated that their work tasks had gradually changed over the years and had become less fulfilling. They had been given more tasks they did not like, (e.g. administrative or computer tasks), leaving less time for the tasks they *did* value (e.g. teaching students or helping patients). They had low job satisfaction and indicated this as an important reason for their sick leave, especially if combined with high work pressure.

[What made the return to work easier] is that I felt connected with my job, I like my job, I have affinity with my job, with the people on my job. I get respected as a person. [Worker with short-term sickness absence: man, 42 years, engineer]

Look, I could return […], but then dissatisfaction would come up again and that is of course one of the factors that why this happened, that it’s not going well. […] I don’t need to be supported [..] It is about that I don’t like it [the job]. [Worker with long-term sickness absence: woman, 44 years, coordinator quality department]

## Discussion

To gain more insight into how long-term absence in workers with CMDs can be prevented, this longitudinal qualitative study focused on 1) workers’ perceived causes of sickness absence, 2) their perceived RTW barriers and facilitators, and 3) differences and similarities between workers with short, medium and long-term sickness absence. A wide variety of perceived causes were found, emphasizing the complexity of the RTW process. However, four predominant themes emerged from the interviews, i.e. (1) high work pressure or other unwanted changes (e.g. in work tasks), (2) poor relationships with supervisors and/or colleagues, (3) worker’s unhelpful thoughts and feelings, especially related to perfectionism and a lack of self-insight, and (4) workers’ ineffective coping behaviors, e.g. avoidant coping and non-assertive behavior (e.g. not setting clear boundaries). Also for barriers and facilitators to RTW a large variation of factors were found, including three important themes: (1) Whether or not any work adjustments were made to facilitate RTW, (2) Poor versus good relationships with supervisors and colleagues, and (3) whether or not adequate occupational health guidance was provided. As for the third research question, although more similarities than differences were found between the three sub-groups, three main differences emerged especially between those on short-term versus long-term sick leave: Respondents in the short-term group more often reported favorable work conditions (e.g. enjoyable work tasks and good relationships), showed more proactive recovery behavior, and did work they valued and which suited them. In contrast, workers with long-term sickness absence seemed to have unfavorable working conditions, showed more reactive behavior and were in need of professional support, and were more dissatisfied with their job.

An interesting finding of this study is that workers believed their sickness absence was caused by high work pressure or other unwanted work changes, rather than by their mental health issues. This finding has several implications. First, it suggests that lowering work pressure is important to reduce and prevent sickness absence. The importance of work pressure as a contributing factor to sickness absence is also highlighted in other studies which investigate work-focused interventions and work adjustment strategies [29–31]. The current study adds to this literature that especially the combination of both *actual* high work pressure and *subjectively* experienced work pressure is experienced as an important cause of sickness absence. Subjective work pressure may be reduced by good supervisor communication skills and support; for example, by regularly talk about how the worker experiences the workload and supporting the worker in setting their limits and more realistic goals if needed.

Second, whereas work pressure is sometimes difficult to reduce (e.g. in healthcare during the COVID-19 pandemic), the findings underline the importance of changes in work tasks and dissatisfaction with work as a risk factor for sickness absence. Especially when there is a high workload, good supervisor communication about what the worker’s values and needs in work are, can lead to more personalized work adjustments. As individuals might value different things and have different needs, this may also lead to better teamwork.

A third implication is that the common presumption that mental health issues *cause* sickness absence, may not be true from the perspective of the worker. This presumption may be related to the *fundamental attribution error*, a well-known phenomenon in social psychology, which refers to the tendency to over-emphasize dispositions and to underemphasize situational influences as causes of behavior in others [32]. Hence, the mental health issue may be seen as part of the worker, and as a result the situational influences (i.e. the role of the workplace stakeholders and context) may be overlooked. This assumption is supported by findings of a recent study, showing that when managers were asked about who was responsible for stress-related sickness absence, they mentioned personality and individual circumstances (e.g. perfectionism, family problems) of sick-listed workers with stress-related complaints, rather than work-related factors [33]. Also, a meta-synthesis on the RTW process of workers with mental disorders concluded that RTW interventions should not only focus on the coping strategies of the worker but also on the workplace and facilitate social integration of the returned worker [14].

The finding that especially in the long-term group, workers did not like their work tasks anymore, and that over the years they gradually grown apart from what they had once valued when choosing their profession, suggests that a ‘reversed job crafting process’ had taken place. Job crafting is a self-initiated, proactive process at work by which workers change elements of their job to optimize the fit between their job demands and personal needs, abilities and strengths [34]. It is a relatively new concept, and intervention studies have produced favorable effects in employee wellbeing and job performance by stimulating job crafting behaviors [35, 36]. Future studies should explore whether more attention to job crafting in the workplace can actually prevent long-term sickness absence in healthy workers [37]. In addition, the fact that no clear differences appeared between the groups in terms of clinical diagnoses suggests that long-term sickness absence was *not* determined by mental illness severity, but by other, mostly work related and/or psychosocial factors. However, as this was a qualitative study with a small sample, this assumption needs to be confirmed in future quantitative studies.

In the present study, the crucial role of the supervisor was highlighted threefold: as a cause for sickness absence, as a barrier to RTW and as a facilitator for RTW. This implies that improved supervisor skills play an important role in sickness absence and the RTW process for which there are several reasons. First, as supervisors are in a superior position, they may provide the worker with support, understanding and autonomy, or conversely with their opposites, which create feelings of stress [38]. Moreover, the supervisor has an important influence on the workplace atmosphere, can provide a positive example for coworkers (with mental health issues) and can promote inclusiveness [39, 40]. Finally, supervisors can be alert to the wellbeing of their staff, which provides opportunities to prevent sickness absence in workers who themselves do not see the signs due to a lack of self-reflection. By having regular conversations with their workers about what they value in work and what their needs are to realize those work values, supervisors can support job crafting and prevent ‘reversed job crafting’ that was found in the long-term group. The finding that managers play a key role in the RTW process and that improved communication is associated with faster RTW has also been found by others [41–43]. However, whereas managers may acknowledge the importance of communication about mental wellbeing, several studies have shown they often feel uncertain about how to best support workers’ mental wellbeing needs [42, 44], and they do not always see it as their responsibility to start the conversation with the worker [45]. Nevertheless, training managers can significantly improve managers' confidence in supporting the mental wellbeing needs of their staff [42], and future studies should investigate if this can prevent sickness absence.

Another factor of importance for successful RTW was adequate supervision and guidance from occupational health professionals. Especially workers with long-term sickness absence may benefit from close supervision and support, also because they tended to be rather passive themselves. Here the occupational physician can play an important role, by using a process-based approach and monitoring the recovery process, intervening when recovery stagnates and strengthen relapse prevention skills and strategies [46, 47].

## Strengths and Limitations

The study has several strengths and limitations. A strength is the large-scaled and prospective study design and the fact that two interviews were held per respondent. Because of this approach, participants could look back at their sickness absence period and were able to reflect on their own RTW *process* which resulted in valuable insights about causes and effect from their perspective. In addition, by comparing the findings of the three groups, novel information was obtained that—especially when confirmed in quantitative research—may lead to more effective future interventions. Another strength was the use of researcher triangulation in data collection and analysis, which enhanced the validity and reliability of the findings.

A limitation of the study is that only the perspective of the worker was explored. Given that RTW is a complex process in which different stakeholders are involved, it would be valuable to adopt a multi-stakeholder perspective and study the views on RTW barriers and facilitators from employers, occupational health professionals and mental health care professionals. Another limitation is that female and higher educated workers were overrepresented in the interviews. However, as is common in qualitative studies, our aim was to generate insight into a complex phenomenon, rather than to produce findings that can be extended to other populations or settings.

## Conclusion

By focusing on the perspective of workers, valuable insight was gained about the process of RTW. The variety of factors that were perceived to cause sickness absence and facilitated or hindered RTW, emphasize the complexity of the RTW process and highlight the need for more personalized RTW strategies. Findings from the present study suggest that employers who want to prevent sickness absence associated with CMDs should show a genuine interest in what their workers value about their work, what their needs are, and proactively seek for opportunities to create work conditions that enable workers to do valuable work. Investing in manager training to improve communication between manager and employee about wellbeing at work is likely to pay off, as managers have a crucial role in worker’s wellbeing and the prevention of sickness absence.

## Supplementary Information

Below is the link to the electronic supplementary material.

Supplementary file1 (DOCX 14 kb)

Supplementary file2 (DOCX 14 kb)

## Data Availability

The datasets generated during and/or analyzed during the current study are available from the corresponding author on reasonable request.
