# Supplementary material for: Barriers and Facilitators for Return to Work from the Perspective of Workers with Common Mental Disorders with Short, Medium and Long-Term Sickness Absence: A Longitudinal Qualitative Study
Source: J Occup Rehabil. 2021 Sep 27;32(2):272–83. doi: 10.1007/s10926-021-10004-9 (PMC9232415; doi:10.1007/s10926-021-10004-9)
Supplement: Supplementary file 1 — Supplementary file1 (DOCX 14 kb) [file 10926_2021_10004_MOESM1_ESM.docx]

**Appendix A.** **INTERVIEW QUESTIONS - FIRST WAVE INTERVIEW**

PART 1: introduction

Comfort participant. For example: ‘We have already talked on the phone, and you told me that you are sick-leave since **. By then we also talked about your complaints. I would like to know more about your sickness absence.’

1. Can you tell me how you are doing and what is going on?
2. How did you function at work when the problems started? What were difficulties? How did you experience this?
3. At this moment, what makes it difficult for you to do your job? How do you experience this?

PART 2: Cause and onset

1. When did the problems start and how did they develop?
2. What was the straw that broke the camel’s back?
3. When you weren’t doing well, did your supervisor know? And your colleagues? What were your reasons to (not) tell your supervisor or colleagues? Were you able to discuss this? How did you experience this?
4. How did you feel about deciding to stay home/call in sick? Could this have been prevented? What does this mean to you? What does this mean to your environment? What do you miss now that you aren’t working?

PART 3: Return

Now we will talk about how you feel about the return to work.

1. At the moment, are you working on the return to work? Do you think about returning?
2. At the moment, which steps are undertaken concerning the return to work? How do you experience the procedure? With whom do you have contact/who are involved? How do you experience the contact? How do you experience the involvement of others?
3. What do you think of all those involved and their mutual cooperation?
4. How important is it to you to return to work? Who do you think plays an important part? What influence do family/friends/partner have? And the physician? The occupational physician? Colleagues and the supervisor? Practitioners like the psychologist? Do goals/values/ambitions that you want to achieve play a role?
5. When do you think you will return to work? Why not longer or shorter?
6. How easy or difficult do you think it will be to return to work? What does it make easy/difficult to return to work. Who could help to facilitate this?
7. If you would be able to return to work in a week, and you could decide yourself what tasks you would do and how you would start, what would you be able/like to do? What do you need to return to work? Who needs to be involved?
8. In your opinion, what do people in your situation mostly need to be able to recover?
9. How can you be supported to return to work? And in what way?
10. What would your ideal way of returning to work look like?
11. Can you tell if you can discuss your situation at work right now?
12. Did I forget to ask something or do you have remarks/supplements?

PART 4: Closure

Thank you for talking to me. How did you feel about doing this interview?
