# Supplementary material for: Barriers and Facilitators for Return to Work from the Perspective of Workers with Common Mental Disorders with Short, Medium and Long-Term Sickness Absence: A Longitudinal Qualitative Study
Source: J Occup Rehabil. 2021 Sep 27;32(2):272–83. doi: 10.1007/s10926-021-10004-9 (PMC9232415; doi:10.1007/s10926-021-10004-9)
Supplement: Supplementary file 2 — Supplementary file2 (DOCX 14 kb) [file 10926_2021_10004_MOESM2_ESM.docx]

Appendix B. **INTERVIEW QUESTIONS – SECOND WAVE INTERVIEW**PART 1: Period until return to work

1. How are you doing? When did you return to work?
2. Do you experience the cause of your absence differently now? Could it have been prevented? How?
3. What did help in the return to work; what went well in this period/what did make the return easy?
4. What didn’t help in the return to work; what went wrong in this period/what did make the return difficult? What would have been needed to return sooner? When in the process should that have happened?
5. Since last interview, what did exactly happen? Did your problems get solved? How? With whom did you have contact? Interventions/actions?
6. Occupational physician
7. Psychologist
8. Physician
9. Other practitioners
10. Private environment (partner, family, friends)
11. Did those actions suffice your needs?
12. Did the actions fit your needs in resolving problems that you experienced at work?
13. Did you receive information (brochures/websites)?
14. How was the contact with your supervisor?
15. How was the contact with your colleagues? Did you often have contact?
16. Were you able to talk with your supervisor/colleagues about your situation? Did they show understanding? Did you feel supported?
17. Did you discuss what you think is important in work? Goals/values/ambitions? What do you miss from work now that you did not (fully) return?
18. Did the employer, occupational physician and the practitioners like psychologist and GP work together?

PART 2: Return to work; moment of the return

1. How has your return been established? Did you succeed in returning?
2. How did you start working? How has that been established? Who took initiative? What role did the occupational physician, GP, other practitioners or family and friends play?
3. Has your work content and/or work tasks been adjusted? Did it help?
4. What did you supervisor (director, team manager) mean/do for you?
5. What did your colleagues mean/do for you?
6. What did you do yourself? To what extent could you provide input in the process? Did you reveal your wishes/worries?
7. What went well; what made it easy to be return at work? What made it difficult to be back at work?
8. When you returned to work, did you exactly tell what was going on?
9. Were you able to talk about your situation at work with your supervisor/colleagues? Did they show understanding? Were you worried about certain things? Did you discuss that?

PART 3: Period after the moment of return

1. What did help when you were already working? What made it difficult to work?
2. Which tasks or work situations are difficult or not possible yet?
3. To what extent can you provide input? Did you discuss your wishes/worries?
4. To what extent do you have decision authority in matters that are important for you at work?
5. Who are supporting you during this period? Occupational physician, GP, supervisor, colleagues? How does that happen?
6. Do they show understanding? Are you able to discuss your situation with your supervisor/colleagues?
7. What do you think of the total return to work process, from the moment you were on sickness absence?
8. How do you see your job in the future (same job, other job)?
9. What would you advise others who are on sickness absence?
10. What have you learned from the total process?

PART 4: Closure
